# Synthesis of Fe and N Co-doped Bi_2_Ti_2_O_7_ Nanofiber with Enhanced Photocatalytic Activity Under Visible Light Irradiation

**DOI:** 10.1186/s11671-016-1610-7

**Published:** 2016-09-08

**Authors:** Bitao Liu, Qionghua Mo, Jiali Zhu, Zhupei Hou, Lingling Peng, Yijia Tu, Qinyi Wang

**Affiliations:** 1 Research Institute for New Materials Technology, Chongqing University of Arts and Sciences, Yongchuan, Chongqing, 402160 China; 2 Material and Energy Department, Southwest University, Beibei, Chongqing, 400700 China; 3 College of Art and Design, Chongqing University of Arts and Sciences, Yongchuan, Chongqing, 402160 China; 4 Chemical Engineering, University of Missouri, Columbia, MO 65211-2200 USA

**Keywords:** Photocatalysts, Semiconductors, Composite materials, Visible light

## Abstract

A series of N-Fe-Bi_2_Ti_2_O_7_ nanofibers were successfully synthesized. The structure, morphology, visible light photocatalytic properties, and the degradation mechanism of N-Fe-Bi_2_Ti_2_O_7_ were investigated. A new phase of Bi_4_Ti_3_O_7_ and smaller band gap could be observed after doing Fe and N into Bi_2_Ti_2_O_7_. It can degrade 66 % MO and 87 % MB in 120 min under visible light irradiation, which is much more than that of pure Bi_2_Ti_2_O_7_. The results indicate that such unique structure could enhance the charge transfer between the nanostructure interfaces and therefore improve its photocatalytic activities.

**Electronic supplementary material:**

The online version of this article (doi:10.1186/s11671-016-1610-7) contains supplementary material, which is available to authorized users.

## Background

In the past decades, industrial pollution introduced by the development of economy has caused lots of troubles. Photocatalyst is regarded as a promising potential solution to these environmental problems [1, 2]. TiO_2_ semiconducting material has been extensively applied in the field of catalysis and considered as one of the best photocatalytic materials due to its strong oxidizing, long-term thermodynamic stability and relative nontoxicity [3]. However, due to its special band structure, the photogenerated electrons and holes in TiO_2_ would undergo a rapid recombination, significantly decreasing the photocatalytic activity in the pollutants’ degradation process [4]. More importantly, due to its large band gap, TiO_2_ can utilize photons in the wavelength range less than 400 nm [5]. Therefore, the critical issue of improving the photocatalytic activity of TiO_2_ is to effectively inhibit the recombination of photogenerated electron-hole pairs and extend the light absorption to the visible light region [6–8].

Recently, many Bi_2_O_3_-TiO_2_ (BTO)-based composites have been drawn much attention for their unique performance in photocatalysis [9–11]. Bi_2_Ti_2_O_7_ was widely studied due to its narrow band gap of 2.6 eV, which means it can absorb the visible light below 480 nm [12]. However, it still needs to improve the photocatalytic efficiency and enlarge the visible light absorbed range to realize indoor application of the photocatalyst [9–12].

In this work, the N- and Fe-doped Bi_2_Ti_2_O_7_ nanofibers were prepared by a simple emulsion electrospinning process. After Fe and N doping, smaller band gap Bi_2_Ti_2_O_7_ and newly Bi_4_Ti_3_O_7_ phase can be observed_._ This would enlarge the light absorbed range and accelerate the separation of the electron-hole pairs; subsequently, the photocatalytic properties were investigated in detail.

## Methods

### Synthesis of BTO Fibers

BTO fibers were prepared similar to the previous report [8]. As a typical progress, 4.26 g tetrabutyl titanate, 6 g DMF, 0.8 g PVP (Mw = 1300000), 6.08 g Bi(NO_3_)_3_•5H_2_O, and FeCl_3_•6H_2_O were mixed and stirred for 12 h. The spinneret diameter was 0.9 mm, and the distance between the tip of the spinneret and the collector is 20 cm. A direct current voltage of 18 kV was maintained during the electrospun process. The as-prepared fibers were maintained at 600 °C for 2 h. Then, the samples were transfer into nitriding furnace maintained at 500 °C for 8 h.

### Instruments

The instruments were similar to our previously work [13]. The X-ray diffraction (XRD) patterns were recorded by a Danton TD-3500 X-ray diffractometer (Cu-Ka radiation, λ = 1.54 Å). Field emission scanning electron microscope (Hitachi, SU-8020) was used to acquire the scanning electron microscopy (SEM) images. Transmission electron microscopy (TEM) micrographs were taken with a JEOL-JEM-2010 (JEOL, Japan, 200 kV). X-ray photoelectron spectroscopy (XPS) analysis was performed on an ESCA Lab MKII X-ray photoelectron spectrometer (Mg Kα). UV-vis absorption spectra of the samples were obtained on a UV-vis spectrophotometer (Hitachi, U-3900), and BaSO_4_ powder was used as the substrate. The photoelectric performance was measured using an electrochemical system (CHI-660B, China). The counter and the reference electrodes were platinum wire and saturated Ag/AgCl, respectively. 0.1 M NaSO_4_ solution was used as electrolyte solution for the measurement, a 150 W Xe arc lamp was utilized as the light source for the photoelectrochemical (PEC) measurement, and the photoresponse was measured at 0.0 V. Electrochemical impedance spectra (EIS) were recorded in the open circuit potential mode and the frequency was range from 100 kHz to 0.01 Hz.

### Photocatalytic Activity Measurement

As reported in our previously work [13], the photocatalytic activities were evaluated under visible light irradiation using a Xe lamp light source with a 420-nm UV cutoff filter. In a typical process, 100 mg of photocatalyst was dispersed in 100-ml methylene blue (MB) and methyl orange (MO) aqueous solution (50 mg L^−1^), respectively. Before irradiation, the solution was stirred for 30 min in the dark to ensure the establishment of adsorption desorption equilibrium. Under light irradiation and stirring, 3-ml solution was taken at every 10 min, followed by centrifugation and filtration to remove the photocatalysts. The concentrations of dye were analyzed on a Varian UV-vis spectrophotometer (Cary-50, Varian Co.).

## Results and Discussion

**Fig. 1:**
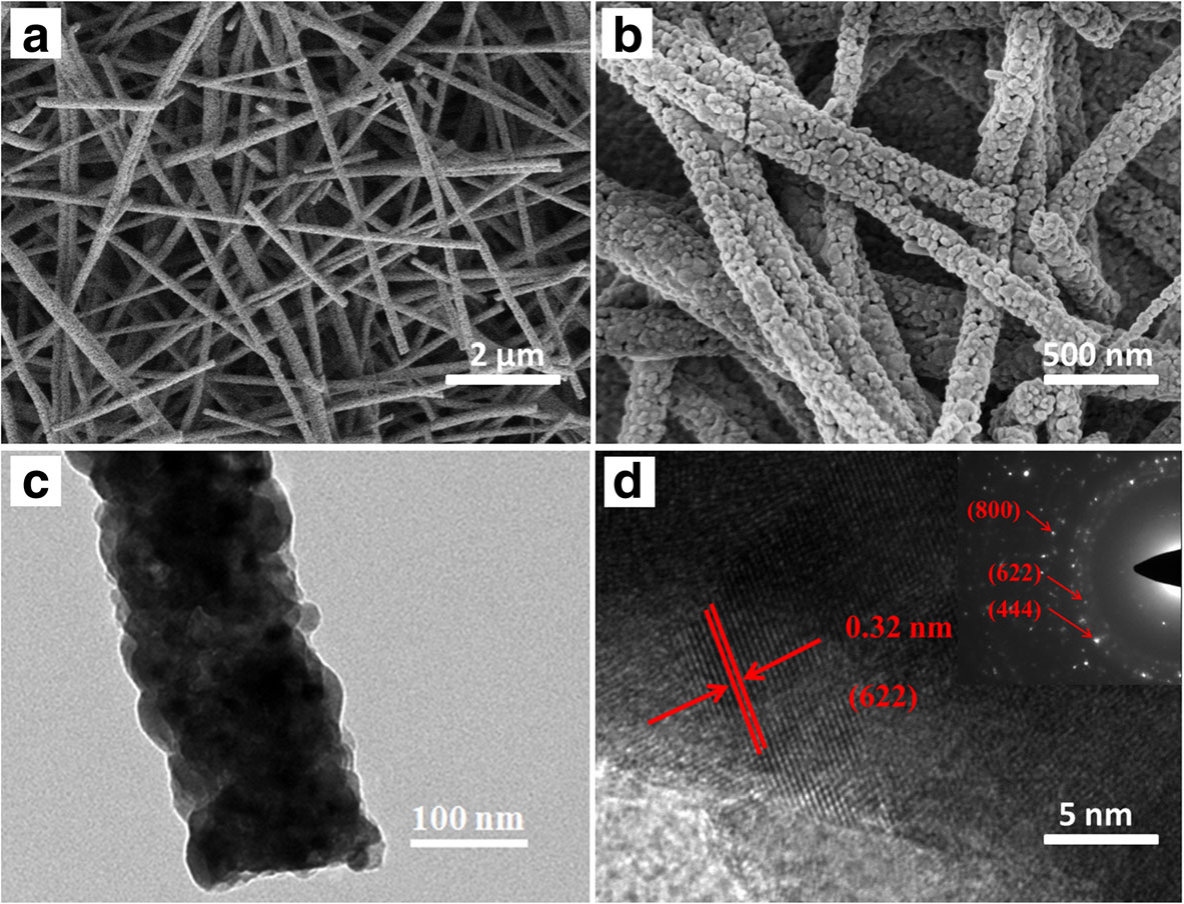
SEM (**a**, **b**) and TEM (**c**, **d**) patterns of N-Fe-BTO 0.5 % samples

The morphology of the N-Fe-BTO 0.5 % samples were shown in Fig. 1. These nanofibers with the diameter of 100~150 nm were consisting with many nanoparticles, as shown in Fig. 1a, b. Obviously, this structure could result in high specific surface area and provide many active sites in the photocatalytic process [8]. And from the TEM images in Fig. 1c, d, clear lattice fringes were observed, indicating that these nanofibers have good crystallinity. The interplanar distances was 0.32 nm, which consisted with the (622) plane of Bi_2_Ti_2_O_7_. Meanwhile, the SAED pattern was also point to the (622), (444), and (800) planes of Bi_2_Bi_2_O_7_.

**Fig. 2:**
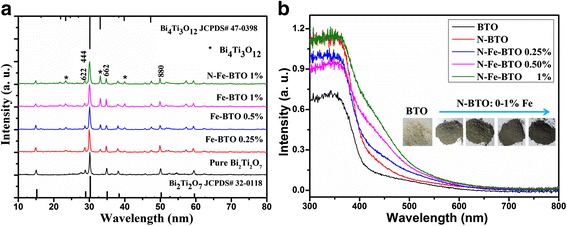
XRD patterns (**a**) and UV-vis diffuse reflectance spectra (**b**) of series samples

The X-ray diffraction patterns of different samples were shown in Fig. 2. It clearly can be seen that a new phase was introduced with the Fe doping, which should ascribe to Bi_4_Ti_3_O_12_ as shown in Fig. 2a. And with the doped Fe increased, the diffraction peaks of Bi_4_Ti_3_O_12_ phase would be enhanced. The structure change also would affect the optical properties, as shown in Fig. 2b. It shows that the absorbance edge extends from 440 to 550 nm, and the color of the powder turns from faint yellow to brownish black with the Fe content increase. This phenomenon was also found in the Fe-doped Bi_2_Ti_2_O_7_ as shown in Additional file 1: Figure S1. Obviously, this red shift should ascribe to the heterostructure between Bi_2_Ti_2_O_7_ and Bi_4_Ti_3_O_12_ phase. Additionally, it also can be seen that the absorbance edge of N-doped Bi_2_Ti_2_O_7_ also shifted to longer wavelength. It means the N doping would be a key factor of the absorption properties, which can introduce an impurity energy level [14].

**Fig. 3:**
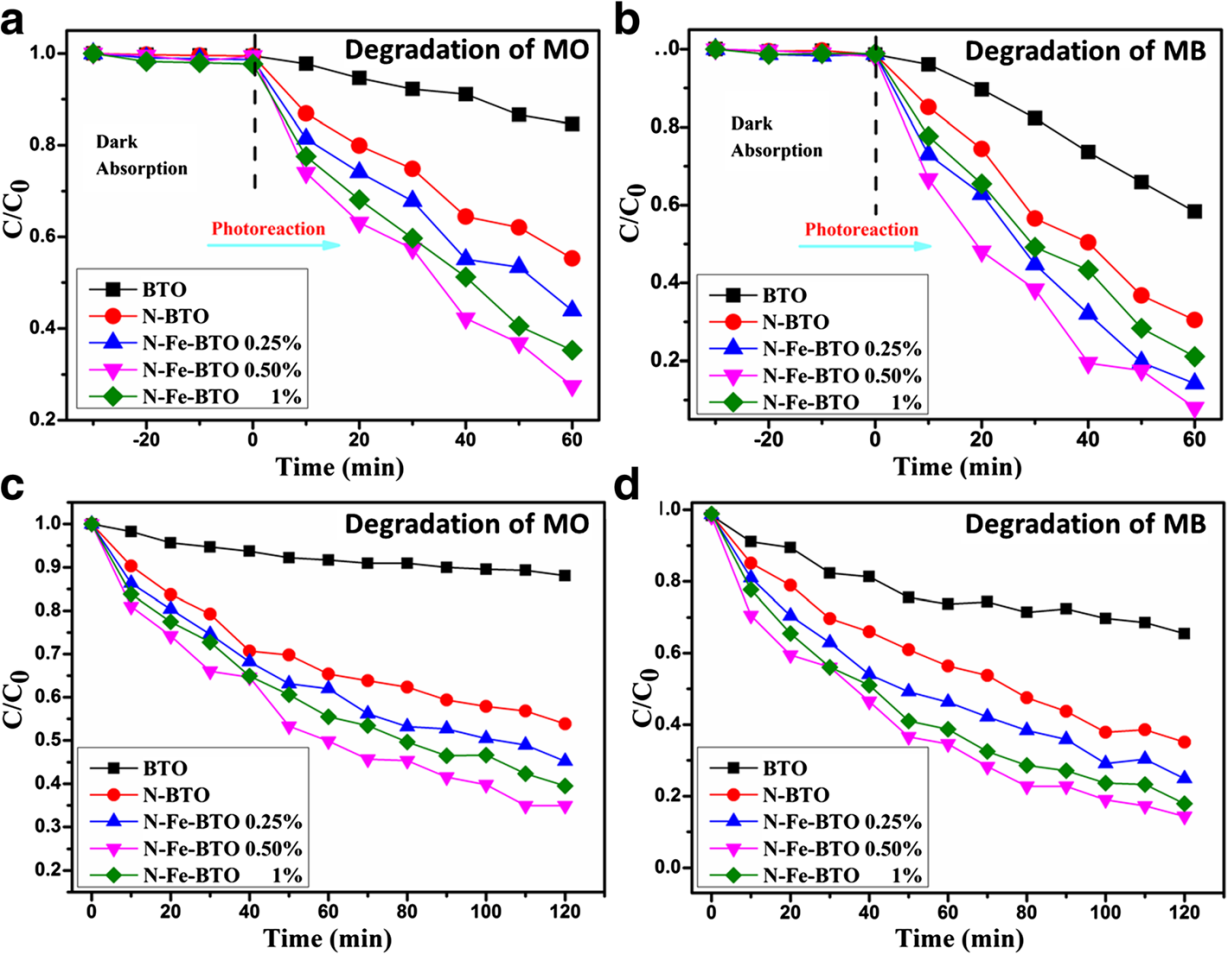
Photodegradation of samples under UV (**a**, **b**) and visible (**c**, **d**) light

The photocatalytic degradation under UV light was listed in Fig. 3a, b. As can be seen, the photocatalytic activity was enhanced with increasing the Fe content. The photocatalytic activity under UV light exhibits a slight decrease if the Fe content was as high as 1 %. The N-Fe-BTO 0.5 % sample exhibited the best photocatalytic activity among all the samples, which degraded 73 % MO and 91 % MB in 60 min while it degraded only 18 % MO and 41 % MB for the pure BTO sample. Additionally, it also shows that the N-BTO exhibited a much better photocatalytic activity than the pure sample, which indicated that the N doping would affect the photocatalytic activity. For the visible light, almost the same tendency was observed; the highest sample would degrade 66 % MO and 87 % MB in 120 min while there were only 12 % MO and 36 % MB for the pure BTO sample. And the N doping also would greatly enhance the photocatalytic activity, as shown in Fig. 3c, d. Obviously, the N doping also plays an important role in the photocatalytic process. Overall, all the samples would prefer to degrade MB much more than MO, which should be due to its different molecular structure with different surface electric properties as shown in Additional file 1: Figure S2 [8].

**Fig. 4:**
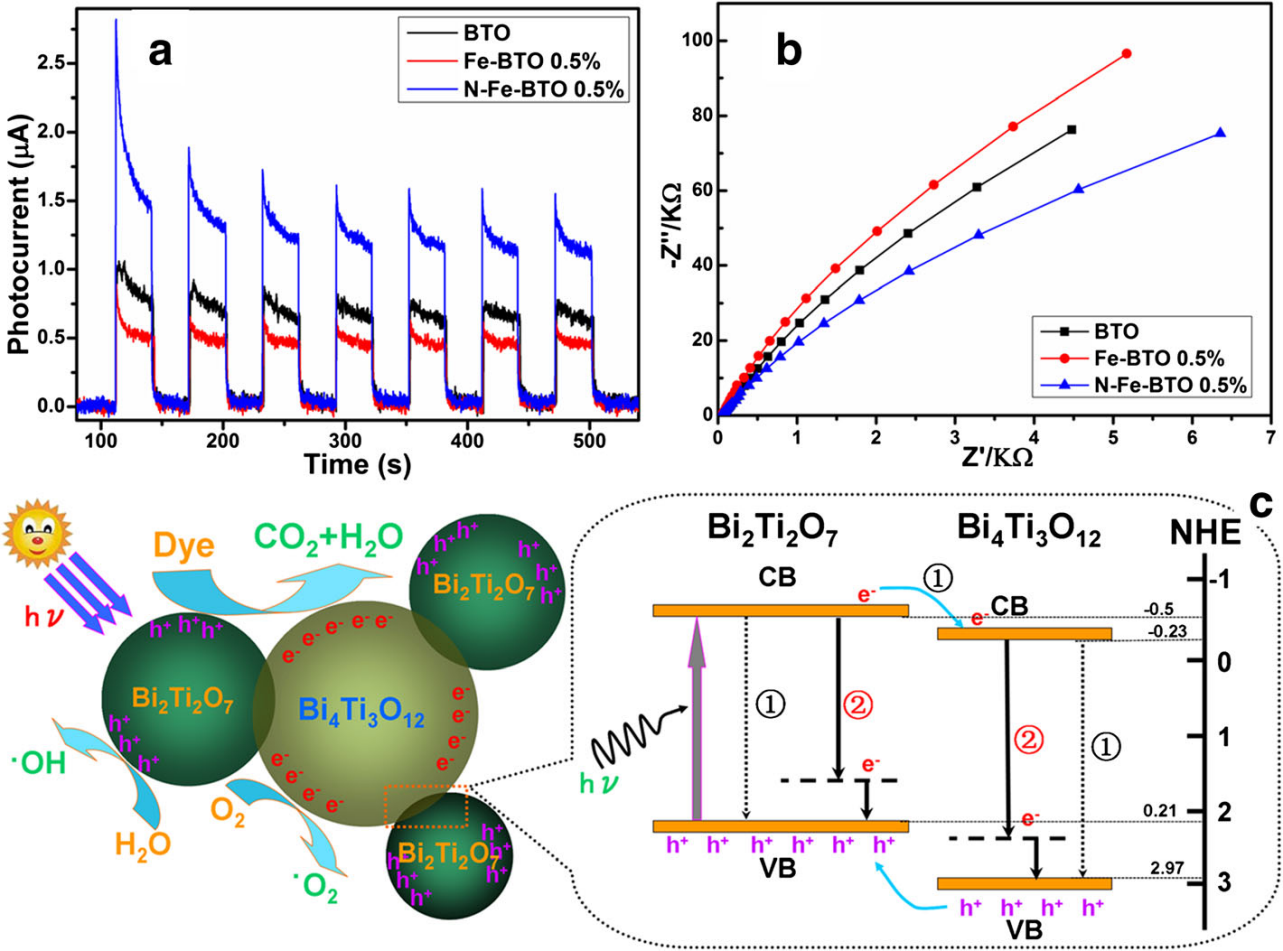
Transient photocurrent responses (**a**), electrochemical impedance spectroscopy (EIS) Nyquist plot (**b**) of the series samples, and schematic mechanism of photocatalytic degradation of organic pollutants under visible light irradiation (**c**)

For a deep investigation, the PEC system was accompanied to investigate the photophysical behaviors under visible light irradiation as shown in Fig. 4a. It was found that the photocurrent response of the Fe-BTO was slightly lower than that of the pure BTO sample, which should be due to the newly introduced Bi_4_Ti_3_O_12_. Though this newly formed heterostructure can separate the electron-hole pairs, it also would remain the photogenerated electrons with a long time due to its huge band gap of 3.2 eV [15]. And for the N doping, it shows that the photocurrent response of N-Fe-BTO was greatly increased. According to the previous work [14], N 2p Orbital electron would affect the valance band and narrow the band gap of TiO_2_; consequently, the N doping would narrow the band gap of BTO and accelerate the electron transfer. The EIS result also proves these results, as shown in Fig. 4b. Thus, a proposed schematic illustration can be shown in Fig. 4c. The Fe doping would result in a new phase of Bi_4_Ti_3_O_12_. This new phase would form a heterostructure and accelerate the electron-hole pair separation as process ① showed. The photogenerated electrons from conduction band of Bi_2_Ti_2_O_7_ to Bi_4_Ti_3_O_12_ would become faster. And with the N doping, the band gap would became narrow; subsequently, the photogenerated electrons would become more efficiently as process ② showed. And then, an enhanced photocatalytic activity can be achieved.

## Conclusions

In summary, N-Fe-Bi_2_Ti_2_O_7_ nanofibers were successfully synthesized by a simple method. It was found that Fe and N doping would play different roles in the photocatalytic process. A newly formed heterostructure between Bi_2_Ti_2_O_7_ and Bi_4_Ti_3_O_12_ and narrow band gap can be achieved. All of this would accelerate the charge transfer and therefore improves its photocatalytic activities.

## Additional File

Additional file 1: Figure S1. UV-vis diffuse reflectance spectra of series samples. **Figure S2.** The molecular structures of the three different dyes. (DOCX 451 kb)
