# Supplementary material for: Synthesis of Fe and N Co-doped Bi2Ti2O7 Nanofiber with Enhanced Photocatalytic Activity Under Visible Light Irradiation
Source: Nanoscale Res Lett. 2016 Sep 8;11(1):391. doi: 10.1186/s11671-016-1610-7 (PMC5016314; doi:10.1186/s11671-016-1610-7)
Supplement: Additional file 1: Figure S1. — UV-vis diffuse reflectance spectra of series samples. Figure S2. The molecular structures of the three different dyes. (DOCX 451 kb) [file 11671_2016_1610_MOESM1_ESM.docx]

**Additional file 1**


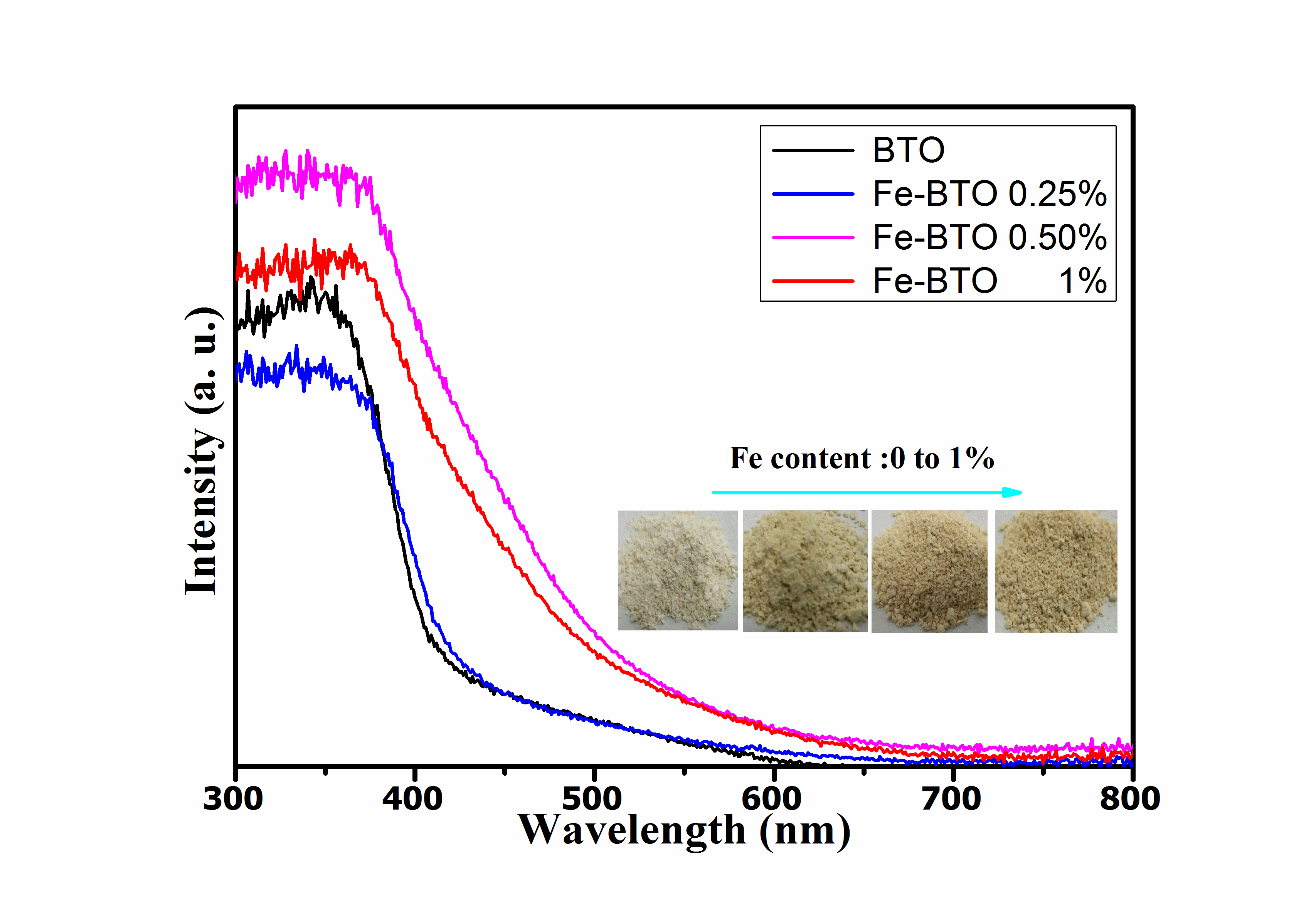


Fig. S1 UV-vis diffuse reflectance spectra of series samples.


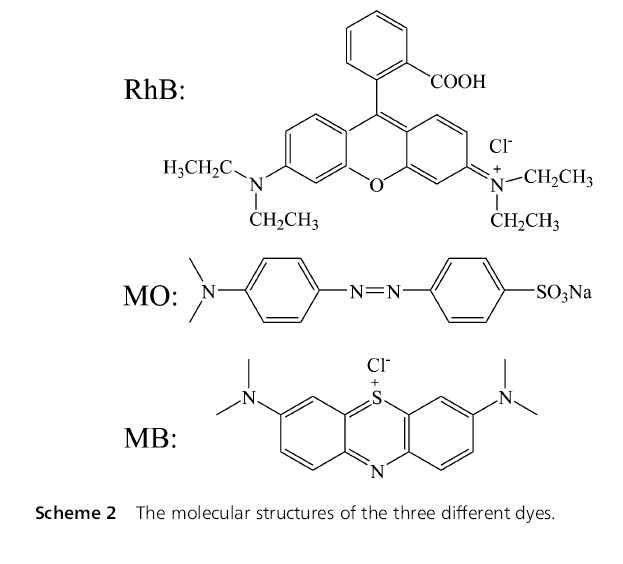


Fig. S2 The molecular structures of the three different dyes.
